# Comparisons of EQ-5D-Y and PedsQL in pediatric patients with mild-to-moderate chronic kidney disease in longitudinal analyses

**DOI:** 10.1186/s12955-023-02197-9

**Published:** 2023-10-27

**Authors:** Chien-Ning Hsu, You-Lin Tain, Pei-Chen Lu, Hsiang-Wen Lin

**Affiliations:** 1 Department of Pharmacy, Kaohsiung Chang Gung Memorial Hospital, Kaohsiung, Taiwan; 2 School of Pharmacy, Kaohsiung Medical University, Kaohsiung, Taiwan; 3 Division of Pediatric Nephrology, Kaohsiung Chang Gung Memorial Hospital and Chang Gung University, Kaohsiung, Taiwan; 4 School of Pharmacy and Graduate Institute, College of Pharmacy, China Medical University, No. 100, Sec. 1, Jingmao Rd., Taichung City, 406040 Taiwan; 5 Department of Pharmacy, China Medical University Hospital, Taichung, Taiwan; 6 Department of Pharmacy Systems, Outcomes & Policy, College of Pharmacy, University of Illinois at Chicago, Chicago, IL USA

**Keywords:** Pediatrics, Chronic kidney disease, Health-related quality of life, EQ-5D-Y, PedsQL, Longitudinal analysis

## Abstract

**Objective:**

To characterize longitudinal changes and correlations between the measures of EQ-5D-Y and generic PedsQL and their associations with clinical changes in children and adolescents with mild-to-moderate chronic kidney disease (CKD).

**Methods:**

Participants were recruited from January 2017 to September 2021 in a medical center in Taiwan. Both instruments were administered in their initial visits and every 6-month subsequent visits. Spearman’s Rho (ρ) was used to assess correlations between the scores of EQ-5D-Y and PedsQL measures in longitudinal changes. Cohen’s effect size (ES) was used to evaluate the changes of scores/subscales over time. In addition, factors associated with longitudinal changes in the score/subscales were explored.

**Results:**

A total of 121 participants were enrolled, and 83 with ≥ 3 HRQOL measures during the 3.5 years follow-up were assessed their changes of HRQOL measures. The correlations (ρ > 0.3) appeared between the changes in the visual analog scale (VAS) of EQ-5D-Y and emotional and social subscales of PedsQL. ES was small (< 0.5) in the VAS and level-sum-score (LSS) of EQ-5D-Y scores for the clinical changes in comorbidities, while some PedsQL subscales were medium to high (0.5–0.8 or > 0.8). Hypertension, mineral bone disorder/anemia, and hyperuricemia associated with the changes in both HRQOL scores were varied by their various domains.

**Conclusion:**

Both EQ-5D-Y and PedsQL of HRQOL measures were responsive to worsened childhood CKD-related comorbidities during the follow-up; however, convergent validity between them was limited in some domains. The LSS of EQ-5D-Y showed greater changes than the VAS by comorbidity status; further comparison with utility weight is needed to determine the better performance of EQ-5D-Y.

**Supplementary Information:**

The online version contains supplementary material available at 10.1186/s12955-023-02197-9.

## Introduction

Chronic kidney disease (CKD) is a worldwide public health problem. Children and adolescents with CKD have an increased risk of premature death from cardiovascular disease and end-stage kidney disease requiring kidney replacement therapy such as dialysis and kidney transplantation. In addition, CKD progression is associated with some complications in the pediatric population, including hypertension, hyperlipidemia, anemia, osteodystrophy, growth retardation, and impaired neurocognitive development [1–3], which may cause a decline in health-related quality of life (HRQOL) in childhood, adolescents and young adulthood [4–6].

Patient-reported outcomes instruments have been used for pediatric CKD, such as generic instruments of the Pediatric Inventory of Quality of Life Scales (PedsQL 4.0) [5, 7–9], the 3-level EuroQol Group's Five Dimensions for Youth (EQ-5D-Y 3L) [10], KIDSCREEN 52 [11], Patient Reported Outcomes Measurement Information System [12, 13], and the Health Utilities Index [14]; and end-stage kidney disease-specific PedsQL3.0 [15, 16]. These studies evaluated the impact of CKD or specific comorbidities of CKD progression on patient-reported outcomes focusing on children and adolescents with moderate or advanced childhood CKD [17]. However, few have evaluated pediatric patients with mild CKD (eGFR ≥ 60 ml/min/1.73 m^2^). Using the Taiwanese version of EQ-5D-Y 3L, our previous work showed that children with mild-to-moderate CKD are able to self-report HRQOL from their own perspective and demonstrated that multiple overt comorbidities were associated with a poor EQ-5D-Y 3L visual analog scale (VAS) [10]. In addition, the changes in CKD comorbidity burden can contribute to a considerable longitudinal discordance in child-parent dyads reported PedsQL scores, particularly in psychosocial domains [18].

Different HRQOL instruments may reflect different theoretical bases, and their content is designed to capture differences in several dimensions, including physical, emotional, mental, and social functioning, in children with chronic illnesses or specific medical interventions [19]. The Taiwan version of EQ-5D-Y has been validated and applied in pediatric kidney [10] as well as PedsQL has been done in pediatric kidney [20] and adiposity [21] populations. Although the PedsQL 4.0 with child self- and parent proxy-report forms has been validated for pediatric CKD [7, 9, 20], it is unknown either PedsQL 4.0 or EQ-5D-Y 3L is a better choice of HRQOL instrument to be regularly used during routine clinical visits in Taiwan yet. This study aimed to compare the psychometric characteristics of EQ-5D-Y and PedsQL Generic score 4.0 in Taiwanese children and adolescents with mild-to-moderate CKD and to investigate potential factors associated with longitudinal changes in HRQOL scores during long-term follow-up.

## Material and methods

### Patients and study settings

We used data from a prospective cohort study of the Precision Medicine Project for pediatric CKD (PMP-PCKD), which was initiated at the end of 2016 in Kaohsiung Chang Gung Memorial Hospital, a tertiary medical center in Taiwan [3, 22, 23]. Those pediatric patients aged 7–18 years who met the inclusion criteria of CKD and were willing to participate were invited to join the study during their routine visits in outpatient settings.

### Measures of HRQOL

The traditional Chinese character of Taiwan version EQ-5D-Y 3L and PedsQL 4.0 instruments were administered to those participants with CKD aged 7–18 years and their parents (mother, father, or relative family) by the trained research coordinator. The standard EQ-5D-Y consists of a descriptive system that includes five dimensions: (1) mobility (“walking about”); (2) self-care (“looking after myself”); (3) usual activities (“doing usual activities”); (4) pain/discomfort (“having pain or discomfort”); and (5) anxiety/depression (“feeling worried, sad, or unhappy”). Each dimension has three ordinal levels to represent its severity. In addition, the sum of all problem level values for the five dimensions to calculate the level-sum-score (LSS) was also used to reflect the severity of different health states [24]. More specifically, the LSS ranges between 5 (health state 11,111 = 1 + 1 + 1 + 1 + 1 = 5) and 15 (health state 33,333 = 3 + 3 + 3 + 3 + 3 = 15) in EQ-5D-Y-3L, with a large LSS indicating a worse health state. The EQ-5D-Y-3L instrument also includes a VAS with the best imaginable health being anchored at 100 and worst imaginable health anchored at 0 to represent the overall health status rated by the participant [25]. All items in EQ-5D-Y-3L refer to the health state for “today”.

PedsQL, a generic instrument, has a parallel child (self) and parent (proxy) forms for children and adolescents aged 5–7, 8–12, and 13–18 years, and a parent report form only for children aged 2–4 years. PedsQL includes 23 items in four domains of physical, emotional, social, and school functioning and provides a total score and its two subscales (Physical Health summary [physical functioning] and Psychosocial Health summary [emotional, social, and school functioning]). Each item was designed to report the problem using a 5-point Likert scale (0: never a problem, 4: always a problem) for a certain condition within the past 30-day period. The reliability and validity of the PedsQL have been confirmed in a pediatric CKD population and other chronic illnesses [7, 26].

### Assessments of HRQOL, clinical health data, and outcomes

HRQOL data for this study were collected at study entry (initial visit) and afterward (usually every 6 months) until patients started their dialysis or turned 18 for their latest follow-up visit. All assessments were completed between January 2017 and September 2021. Each child completed both EQ-5D-Y and PedsQL independently in a private space next to the physician’s consulting room in an outpatient setting. Those primary caregiver or other relatives who can provide adequate information about their child’s HRQOL during the outpatient care visit were invited to assess the PedsQL parent proxy version.

Clinical health data related to CKD were collected at the initial visit and at the same time when performing each HRQOL measurement assessments, as described in previous studies [3, 22, 23]. To evaluate the responsiveness of HRQOL measurements across times for CKD pediatric patients, their clinical changes were categorized as “no change”, “improved” (the number of comorbidities decreased), and “worsened” (the number of comorbidities increased) based on the number of comorbid conditions at the time of initial visit (T1) and the latest visit (T2) in the follow-up, same as our previous work [18]. Eight common pediatric CKD comorbid conditions, which were pre-specified as growth retardation (body height or body weight < 3^rd^ percentile), overweight (body mass index > 90^th^ percentile), hypertension (systolic or diastolic blood pressure > 95^th^ percentile), hyperlipidemia (total cholesterol > 200 mg/dL, triglycerides > 95^th^ percentile, or low density lipoprotein > 130 mg/dL), mineral bone disorders (serum phosphate multiplied with serum calcium ≥ 6.5 [≤ 12 years], ≥ 5.5[> 12 years]), anemia (hemoglobin < 5^th^ percentile), hyperuricemia (serum uric acid > 5.9 mg/dL), and proteinuria (urine total protein divided by urine creatinine > 150 mg/g) [3, 18, 22, 23] were also evaluated and compared. Comorbid conditions were based on age- and sex-specific percentile norms in the pediatric population. Kidney function was assessed by determining estimated glomerular filtration rate (eGFR) from serum creatinine (SCr) using the Schwartz bedside Eq. (0.413 × height [cm]/ SCr [mg/dL]) [27]. For the individual CKD comorbid condition, yes-yes (persistent) indicated the specific commodity presented at T1 ( +) and T2 ( +) visits, no-yes (developed) as T1 (-) and T2 ( +), yes–no (improved) as T1 ( +) and T2 (-), and no–no (no change) as T1 (-) and T2 (-).

### Statistical analyses

**Fig. 1:**
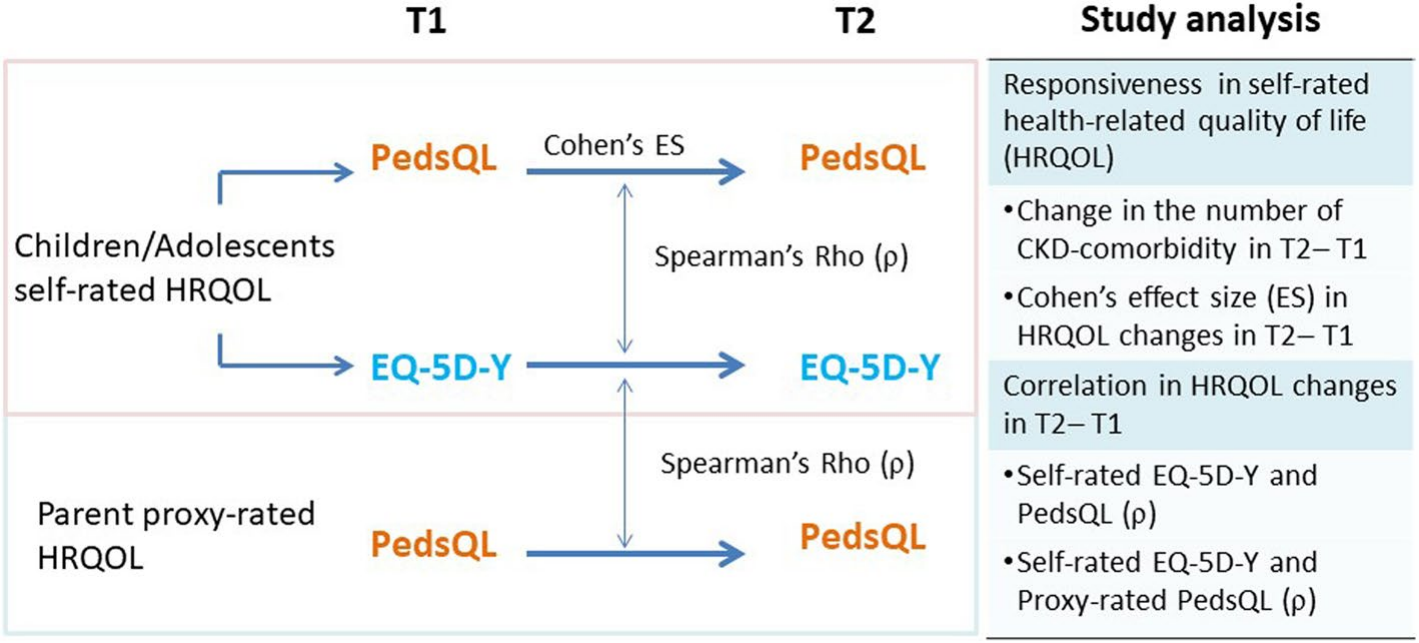
Analysis and comparison between EQ-5D-Y and PedsQL measures. T1: the initial (baseline) assessment; T2: the latest follow-up assessment

Continuous data were presented as means with standard deviations, and categorical data were presented as numbers and percentages. All enrolled pediatric patients were grouped as children (in elementary school ages of 7–12 years-old) and adolescents of 13–18 years-old to compare their characteristics. We assumed that a strong enough correlation between child self-rated EQ-5D-Y and PedsQL reports was existed in this study. Since the VAS of EQ-5D-Y did not follow a normal distribution, non-parametric Spearman’s Rho (ρ) correlation tests were used to assess the convergent validity between EQ-5D-Y and PedsQL difference from T1 toT2. The correlation coefficients of 0.3 or above were considered as evident correlation [28]. Figure 1 presents the analysis plan of this study. Cohen’s effect size (ES) was applied to measure the responsiveness of HRQOL in clinical changes (defined calculation approach). The paired *t*-test statistics and ES of change in the VAS and LSS of EQ-5D-Y and PedsQL scores were used for individual-level analysis between T1 and T2. According to Cohen, ES of 0.8 or above was considered large, 0.5 to 0.8 medium, 0.2 to 0.5 small, and  < 0.2 was trivial [29].

A linear mixed-effect model was used to assess the magnitude of changes in summary scores of EQ-5D-Y, PedsQL and its subscales between T1 and T2 (T2-T1), adjusted for age, gender, type of CKD, and 4-level changes in each comorbidity status between T1 and T2 (no–no [reference], yes–no, no-yes, and yes-yes). The results are presented as estimates of beta (β) with 95% confidence intervals (CI). All analyses were performed using SAS version 9.4 (SAS Institute Inc., Cary, NC, United States). The criterion for statistical significance was a two-tailed *p*-value of < 0.05.

## Results

### Patient characteristics

A total of 121 children and adolescent responded to both EQ-5D-Y and PedsQL measures in their initial visits. Of them, 83 had ≥ 3 measures of HRQOL and comprehensive laboratory results in 485 patient visits during the 3.5 years follow-up. Forty-five (54%) patients had ≥ 6 time points, 30 patients (36%) had 4–5 time points and 8 patients had 3 time points of assessment were included in the responsiveness analysis of HRQOL about their clinical changes. (1.5 years) Given they were averaged 11.47 (± 3.44) years-old, 57% were boys, 78.8% had at least one common comorbidity (Table 1). Totally, fifty-four percent of children/adolescents were diagnosed with congenital anomalies of the kidney and urinary tract (CAKUT) and 31.41% had their estimated glomerular filtration rate (eGFR) less than 89 ml/min/1.73 m^2^.While 56% (*n*  =  68) and 44% (*n*  =  53) of them were in the elementary or high school ages, respectively, those child in high school tended to had more severe renal function, overweight, comorbidities of bone disorders/anemia, hyperuricemia and proteinuria and more number of comorbid, than younger children.

**Table 1:** Characteristics of pediatric patients in their initial visits

Variable	All pediatric patients	7–12 years	13–18 years	*p value*^a^
	*n* = 121	*n* = 68	*n* = 53	
Sex, n (%)				0.9342
Boy	69 (57.02)	39 (57.35)	30 (56.60)	
Girl	52 (42.98)	29 (42.65)	23 (43.40)	
Type of CKD				0.0616
CAKUT: Renal agenesis/hypoplasia	46 (38.02)	33 (48.53)	13 (24.53)	
CAKUT: others	19 (15.70)	9 (13.24)	10 (18.87)	
Glomerular disease	27 (22.31)	13 (19.12)	14 (26.42)	
Others	29 (23.97)	13 (19.12)	16 (30.19)	
eGFR at baseline, ml/min/1.73m^2^,group, n (%) (*n* = 120)				
≥ 90	82 (67.77)	52 (77.61)	30 (56.60)	0.0428
60–89	26 (21.49)	11 (16.42)	15 (28.30)	
30–59	12 (9.92)	4 (5.97)	8 (15.09)	
Comorbid conditions at baseline, n (%)				
Growth retardation (*n* = 120)	19 (15.83)	9 (13.43)	10 (18.87)	0.4180
Overweight (*n* = 119)	36 (30.25)	15 (22.39)	21 (40.38)	0.0340
Hypertension (*n* = 118)	45 (38.14)	21 (31.82)	24 (46.15)	0.1114
Hyperlipidemia (*n* = 118)	20 (16.95)	11 (16.67)	9 (17.31)	0.9266
Mineral bone disorders/Anemia (*n* = 120)	16 (13.33)	3 (4.48)	13 (24.53)	0.0013
Hyperuricemia (*n* = 120)	28 (23.33)	8 (11.94)	20 (37.74)	0.0009
Proteinuria (*n* = 119)	44 (36.97)	19 (28.36)	25 (48.08)	0.0271
Number of comorbid conditions, n (%) (*n* = 118)				0.0011
None	25 (21.19)	19 (28.79)	6 (11.54)	
1	29 (24.58)	19 (28.79)	10 (19.23)	
2	36 (30.51)	21 (31.82)	15 (28.85)	
≥ 3	28 (23.73)	7 (10.61)	21 (40.38)	

On average, pediatric patient’s age was 11.47 (3.44) years old at study enrolment

*CKD* chronic kidney disease, *CAKUT* Congenital anomalies of kidney and urinary tract, *eGFR* Estimated glomerular filtration rate

^a^Comparison of different age group by Chi-square test or Fisher’s exact test for categorical data

### HRQOL measures and comorbid conditions in their initial visits

Table 2 presents median summary scores of EQ-5D-Y and PedsQL in their initial visits by the number of baseline comorbidities. In general, both instruments can discriminate those patients’ HRQOL with ≥ 3 comorbid conditions for their lower scores of VAS of EQ-5D-Y and total score of PedsQL than those with less comorbid conditions. Among those patients with ≥ 3 comorbid conditions (n = 28), the median VAS of EQ-5D-Y was 95 (IQR: 90–99.8), and the total score of PedsQL was 89.8 (IQR, 87.0–97.3).

**Table 2:** Distribution of HRQOL scores by number of comorbidities in their initial visits among all enrolled pediatric patients

Scores/subscales of HRQOL	All pediatric patients (*n* = 121)	Number of comorbidities			
		None (*n* = 25)	with 1 (*n* = 29)	with 2 (*n* = 36)	with ≥ 3 (*n* = 28)
EQ-5D-Y VAS	97.0 (90.0, 100.0)	97.0 (90.0, 100.0)	97.0 (90.0, 100.0)	100.0 (92.5, 100.0)	95.0 (90.0, 99.8)
EQ-5D-Y LSS	5.0 (5.0, 6.0)	5.0 (5.0, 5.0)	5.0 (5.0, 5.0)	5.0 (5.0, 5.0)	5.0 (5.0, 5.0)
PedsQL					
Total Score	93.4 (86.3, 97.5)	94.4 (86.6, 96.3)	93.4 (84.4, 100.0)	91.3 (84.3, 97.5)	89.8 (87.0, 97.3)
Physical Health	96.9 (87.5, 100.0)	93.8 (87.5, 100.0)	96.9 (87.5, 100.0)	100.0 (87.5, 100.0)	93.8 (89.1, 100.0)
Emotional	95.0 (80.0, 100.0)	90.0 (75.0, 100.0)	100.0 (75.0, 100.0)	95.0 (82.5, 100.0)	95.0 (77.5, 100.0)
Social	100.0 (95.0, 100.0)	100.0 (95.0, 100.0)	100.0 (95.0, 100.0)	100.0 (95.0, 100.0)	100.0 (95.0, 100.0)
School	90.0 (75.0, 95.0)	90.0 (80.0, 95.0)	90.0 (80.0, 100.0)	80.0 (75.0, 95.0)	85.0 (75.0, 95.0)

Values presented as median (25^th^, 75^th^) by numbers of comorbidity at study enrolment

*VAS* Visual Analogue Scale, *LSS* Level Sum Score, A higher LSS indicated a worse health state

In their initial visits (T1), a very few participants reported “some problems” and “a lot of problems” for the EQ-5D-3L-Y descriptive system and all participants had no problems in “self-care” and “usual activities”. The younger children tended to have any problems on pain/discomfort or anxiety/depression domains but adolescent children reported worse EQ VAS (Table S1). The highest LSS was 8 (*n*  =  1 with 21,122), followed by LSS of 7 (*n*  =  4) and 6 (*n*  =  20). The LSS of EQ-5D-Y appeared similar across all the comorbid condition groups. Children/adolescents had a worst health state (LSS > 5) rated a lower VAS score in their initial visits (Table S2). Higher LSS (ranged 6–7) gave some indications that children/adolescents may rate VAS score differently depending on the dimensions of mobility, pain/discomfort and anxiety/depression.

### Convergent validity (correlations between EQ-5D-Y and PedsQL)

For the longitudinal changes in the VAS of EQ-5D-Y, there were few significant correlations (ρ > 0.3), including the changes in the total score and emotional and social domains of patients' self-rated PedsQL (Table 3). Low correlation was observed between “anxiety/depression” of EQ-5D-Y and emotional PedsQL score (ρ*,* -0.24, *p* = 0.0203). Cross-sectional correlation coefficients between EQ-5D–Y and PedsQL subscales in their latest follow-up visits (T2) were generally higher (ρ > 0.3) than the longitudinal correlations among a subgroup of children/adolescents in the longitudinal analyses (Tables S3 and 3). Nevertheless, correlations between parent proxy-reported PedsQL scores and child self-reported EQ-5D-Y were also very small (ρ < 0.2 in Table S4).

**Table 3:** Correlations between the changes of EQ-5D-Y and PedsQL scores across times among who completed both initial and latest follow-up assessments (*n* = 83)

	Absolute difference in summary scores of EQ-5D-Y				Change in each dimension of EQ-5D-Y							
Difference in PedsQL	VAS		LSS		Mobility		Usual activities		Pain/ discomfort		Anxiety/ depression	
	ρ	*p*—value	ρ	*p*—value	ρ	*p*—value	ρ	*p*—value	ρ	*p*-value	ρ	*p*-value
Total Score	0.32	0.0020	-0.13	0.2141	-0.17	0.1039	-0.18	0.0866	-0.01	0.9353	-0.16	0.1341
Physical Health	0.17	0.1171	-0.15	0.1483	-0.16	0.1215	-0.17	0.1102	-0.07	0.4916	-0.15	0.1695
Emotional	0.32	0.0019	-0.12	0.2405	-0.10	0.3332	-0.19	0.0784	0.09	0.4184	-0.24	0.0203
Social	0.32	0.0023	-0.08	0.4341	-0.15	0.1543	-0.21	0.0504	0.03	0.7681	-0.01	0.9408
School	0.26	0.0151	-0.16	0.1376	-0.19	0.0691	-0.17	0.1030	-0.11	0.2854	-0.06	0.5750

“Self-care” of EQ-5D-Y is not presented due to the no patients reported any problems at initial (T1) and the latest follow-up assessments (T2)

Difference = T2 score minus T1 score; 3-level change in each dimension of EQ-5D-Y, including improve (T1: having any problem, T2: no problem), no change (T1 and T2 same), and worsen (T1: no problem, T2: having any problem), Supplementary Table S3 presents changes in each comorbidity status

ρ (Rho): Spearman’s correlation coefficient, positive correlation coefficient indicates agreement between measures; negative correlation coefficient indicates disagreement between measures

*VAS* Visual Analogue Scale, *LSS* Level Sum Score, A higher LSS indicated a worse health state

### Responsiveness

**Fig. 2:**
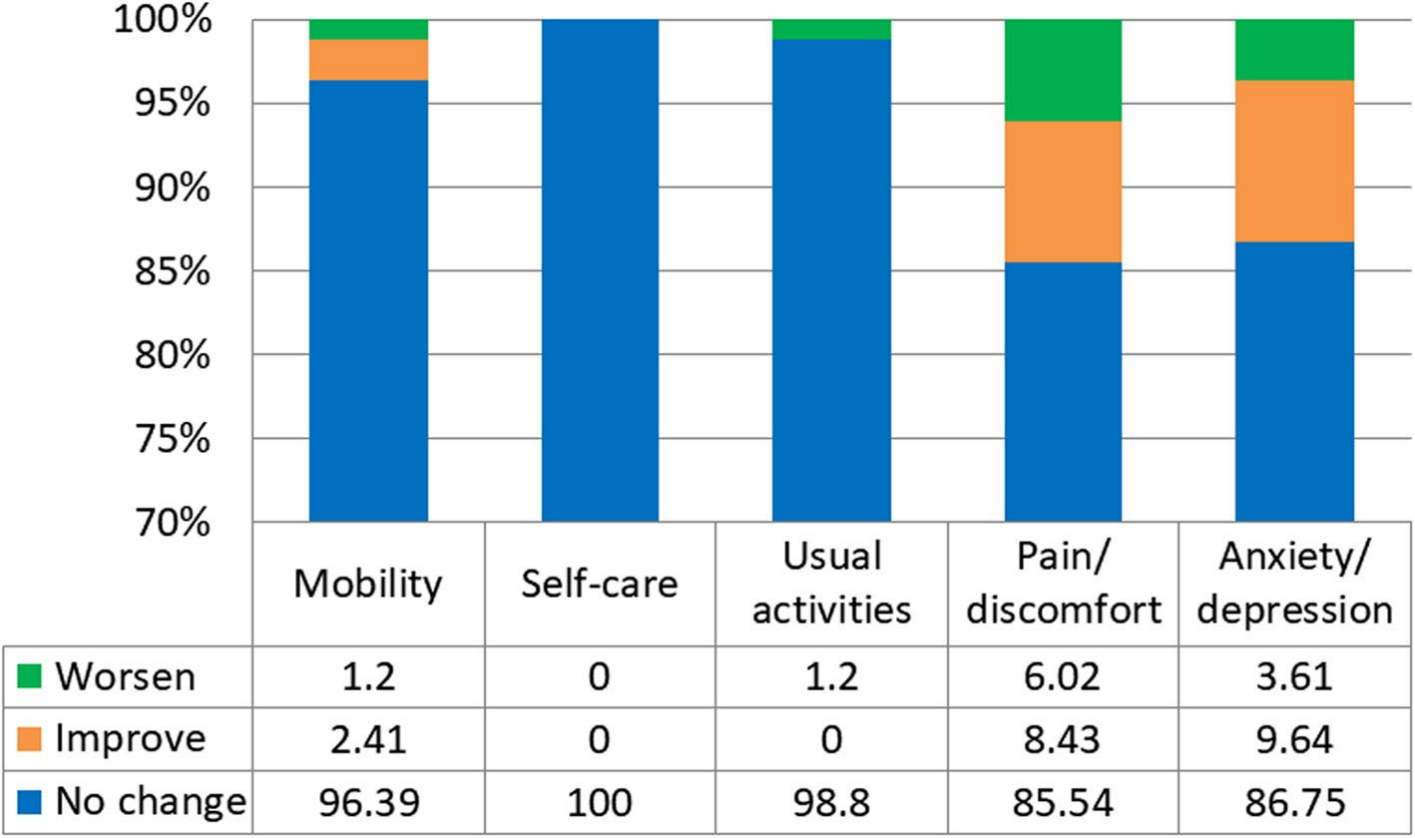
The proportion of patients by a change in each dimension of EQ-5D-Y during the follow-up period (*n* = 83); Change: T2 (the latest visit assessment) compared to T1 (the initial visit assessment) for “have any problem” (yes); Worsen (T2:yes, T1:no); Improve (T2:no, T1:yes); No change (T1 = T2)

Figure 2 presents the distribution of EQ-5D-Y domains by the level of clinical changes in the latest follow-up visit. Most (> 96%) of children and adolescents self-reported no changes in “mobility”, or “usual activities” dimensions of EQ-5D-Y. In their follow-up visits, approximately 15% of participants reported the changes either better or worse or have any problems (vs. no problems) in pain/discomfort” (14.45%) and “anxiety/depression” (13.25%) of EQ-5D-Y.

Table 4 presents the longitudinal differences in HRQOL by the level of changes in CKD-related comorbidity over the study period. The ES was small (0.2–0.5) for the VAS and LSS of EQ-5D-Y by the classification of clinical changes (no change, improvement, and worsening). The ES was large for the total score of PedsQL, corresponding to improved and worsened CKD-related comorbidities (ES > 0.8). The distribution of clinical changes indicated that more patients in the worsened status group had multiple worsened comorbid conditions (i.e., hypertension, hyperlipidemia, mineral bone disorders/anemia, and hyperuricemia) during the follow-up compared to the other health status groups (no change and improved) (Table S5).

**Table 4:** Changes of HRQOL total scores/subscales by the classification of clinical changes in the number of CKD-related comorbidity among who completed both initial and latest follow-up assessments (*n* = 83)

Changes of HRQOL total scores/subscales		Changes on number of CKD-related comorbidities (T2-T1)			
	All (*n* = 83)	No change (*n* = 34)	Improved (*n* = 16)	Worsen (*n* = 33)	*p* value*
	mean (SD)	mean (SD)	mean (SD)	mean (SD)	
**Mean difference (T2-T1)**					
EQ-5D-Y VAS	-1.10 (11.05)	-4.00 (11.69)	-2.50 (9.31)	2.56 (10.39)	0.0427
EQ-5D-Y LSS	-0.08 (0.65)	0.03 (0.72)	-0.06 (0.68)	-0.21 (0.55)	0.3124
PedsQL					
Total score	5.61 (8.15)	4.06 (8.99)	6.43 (6.22)	6.81 (8.03)	0.3546
Physical Health	5.57 (9.40)	3.31 (7.57)	8.20 (12.44)	6.63 (9.20)	0.1626
Emotional	7.47 (14.00)	5.74 (14.15)	7.19 (12.11)	9.39 (14.83)	0.5678
Social	2.89 (9.76)	2.35 (11.23)	2.19 (8.94)	3.79 (8.66)	0.7964
School	6.51 (12.27)	4.85 (13.23)	8.13 (10.47)	7.42 (12.19)	0.5880
**Cohen’s effect size**					
EQ-5D-Y VAS	-0.10	-0.34	-0.27	0.25	
EQ-5D-Y LSS	-0.13	0.04	-0.09	-0.39	
PedsQL					
Total score	0.69	0.45	1.03	0.85	
Physical Health	0.59	0.44	0.66	0.72	
Emotional	0.53	0.41	0.59	0.63	
Social	0.30	0.21	0.24	0.44	
School	0.53	0.37	0.78	0.61	

No change: number of comorbidities was same at study enrollment (T1) and the latest follow-up visit (T2); improve = number of comorbidities at T1 > T2; worsen = number of comorbidities at T1 < T2. Cohen’s ES of 0.8 or above was considered large, 0.5 to 0.8 medium, 0.2 to 0.5 small, and < 0.2 was trivial in meaningful changes

*VAS* Visual Analogue Scale, *LSS* Level Sum Score, A higher LSS indicated a worse health state

^a^ Comparison of different clinical changes groups by ANOVA test

### Factors associated with responsiveness of HRQOL

Seven linear mixed-effect models were performed to explore the associations between clinical changes and longitudinal changes for the individual HRQOL total or subscale scores. Overall, differences in children’s self-reported EQ-5D-Y and some domains of PedsQL scores were significantly associated with changes in some specific CKD-related comorbidities. The status changes in overweight, hypertension, and hyperuricemia were consistently associated with responsiveness across the summary scores of VAS and LSS of EQ-5D-Y and the total score of PedsQL over time (Tables 5 and 6). For instance, the EQ-5D-Y VAS was responsive to children/adolescents with persistent and developed overweight, developed hypertension, and developed hyperlipidemia (all *p* < 0.05). Further, adolescents (13–18 years) were associated with decreased VAS of EQ-5D-Y scores over time. The responsiveness of the LSS of EQ-5D-Y was also associated with the baseline number of CKD-related comorbidities, changes in growth retardation, and proteinuria status. The responsiveness of the total score of PedsQL was also significantly associated with mean declined eGFR and mineral bone disorders/anemia.

**Table 5:** Factors associated with the changes of EQ-5D-Y and PedsQL scores/subscales among those who completed both initial and latest follow-up assessments across times (T2-T1) (*n* = 83)

Variables	EQ-5D-Y ΔVAS				EQ-5D-Y ΔLSS				PedsQL ΔTotal Score			
	β*	95% CI		*p-value*	β*	95% CI		*p-value*	β*	95% CI		*p-value*
Intercept	92.07	86.62	97.52	< .0001	5.18	4.97	5.40	< .0001	95.75	91.62	99.88	< .0001
Time (every 6 months)	0.26	-0.08	0.60	0.1404	-0.03	-0.05	-0.01	0.0016	0.81	0.57	1.04	< .0001
Aged 13–18 years (vs 7–12)	-4.98	-7.60	-2.35	0.0004	-0.05	-0.15	0.05	0.3064	0.63	-1.36	2.62	0.5296
Boys (vs Girls)	-1.04	-3.33	1.26	0.3686	0.07	-0.02	0.16	0.1084	-1.13	-2.88	0.61	0.1984
Baseline eGFR	0.02	-0.02	0.06	0.2643	0.00	0.00	0.00	0.2958	-0.02	-0.05	0.01	0.1392
**Change in eGFR, per month**	**-1.15**	**-2.35**	**0.05**	**0.0596**	0.04	-0.01	0.09	0.1584	**-1.50**	**-2.41**	**-0.60**	**0.0015**
Number of comorbidities at T1(vs none)												
1	0.31	-4.18	4.80	0.8892	**-0.28**	**-0.45**	**-0.10**	**0.0023**	-0.71	-4.12	2.69	0.6762
2	-2.30	-9.81	5.22	0.5428	**-0.47**	**-0.76**	**-0.17**	**0.0024**	-2.99	-8.69	2.72	0.2985
≥ 3	-10.84	-23.00	1.32	0.0794	**-0.57**	**-1.07**	**-0.07**	**0.0252**	-7.28	-16.47	1.90	0.1177
Change in comorbidity (vs none at T1 &T2)												
**Growth retardation**												
baseline ( +) & latest (-)	5.65	-1.12	12.41	0.1000	-0.01	-0.27	0.25	0.9236	3.92	-1.24	9.07	0.1334
baseline ( +) & latest ( +)	-0.45	-6.46	5.55	0.8805	0.30	0.06	0.54	0.0148	-1.62	-6.16	2.92	0.4782
baseline (-) & latest ( +)	-0.66	-4.93	3.62	0.7590	0.18	0.02	0.34	0.0283	-2.28	-5.55	0.99	0.1674
**Overweight**												
baseline ( +) & latest (-)	2.35	-3.91	8.62	0.4551	0.28	0.02	0.53	0.0340	3.33	-1.39	8.06	0.1632
**baseline ( +) & latest ( +)**	**6.32**	**2.05**	**10.59**	**0.0045**	0.11	-0.06	0.28	0.1853	**4.77**	**1.54**	**8.01**	**0.0046**
baseline (-) & latest ( +)	4.05	-0.01	8.11	0.0506	-0.05	-0.21	0.11	0.5184	1.14	-1.96	4.24	0.4637
**Hypertension**												
baseline ( +) & latest (-)	2.74	-2.70	8.18	0.3176	0.22	0.00	0.43	0.0457	2.34	-1.80	6.48	0.2626
baseline ( +) & latest ( +)	-2.09	-6.48	2.29	0.3426	0.18	0.00	0.35	0.0525	-0.68	-3.99	2.64	0.6847
**baseline (-) & latest ( +)**	**-4.00**	**-6.84**	**-1.15**	**0.0069**	-0.03	-0.14	0.08	0.5755	**-2.22**	**-4.38**	**-0.06**	**0.0444**
**Hyperlipidemia**												
baseline ( +) & latest (-)	7.91	-0.74	16.55	0.0723	-0.08	-0.45	0.29	0.6632	-0.49	-6.99	6.01	0.8809
**baseline ( +) & latest ( +)**	0.75	-3.80	5.30	0.7425	**0.37**	**0.20**	**0.54**	** < .0001**	0.05	-3.41	3.51	0.9766
baseline (-) & latest ( +)	6.02	1.17	10.87	0.0160	0.06	-0.12	0.24	0.5361	2.45	-1.23	6.14	0.1879
**Mineral bone disorders/Anemia**												
baseline ( +) & latest (-)	4.48	-1.49	10.46	0.1381	0.22	-0.01	0.45	0.0552	-1.13	-5.67	3.42	0.6214
**baseline ( +) & latest ( +)**	-1.88	-8.45	4.69	0.5678	0.20	-0.06	0.46	0.1263	**-6.45**	**-11.43**	**-1.47**	**0.0121**
baseline (-) & latest ( +)	0.42	-4.01	4.86	0.8491	-0.08	-0.25	0.10	0.3904	-3.40	-6.77	-0.03	0.0483
**Hyperuricemia**												
baseline ( +) & latest (-)	2.65	-3.03	8.34	0.3538	0.29	0.07	0.52	0.0111	-0.28	-4.59	4.03	0.8962
baseline ( +) & latest ( +)	2.38	-2.54	7.29	0.3366	0.24	0.04	0.44	0.0176	1.29	-2.43	5.00	0.4899
**baseline (-) & latest ( +)**	1.40	-1.85	4.65	0.3913	-0.04	-0.17	0.09	0.5298	**-3.41**	**-5.88**	**-0.94**	**0.0078**
**Proteinuria**												
baseline ( +) & latest (-)	-1.07	-6.27	4.13	0.6823	0.47	0.26	0.68	< .0001	-2.31	-6.25	1.63	0.2449
baseline ( +) & latest ( +)	1.94	-2.79	6.68	0.4141	0.18	0.00	0.37	0.0514	3.01	-0.58	6.60	0.0984
baseline (-) & latest ( +)	4.45	-0.83	9.74	0.0971	0.00	-0.21	0.20	0.9763	1.49	-2.53	5.51	0.4600

Δ: T2-T1 scores; β*: adjusted regression coefficient in the linear mixed model; positive value in the LSS of EQ-5D-Y model, indicating the comorbid status (vs no–no at T1 &T2) was associated with increased LSS (worsen health states), increased VAS of EQ-5D-Y and increased PedsQL scores indicated improved health

*VAS* Visual Analogue Scale, *LSS* Level Sum Score, A higher LSS indicated a worse health state

**Table 6:** Factors associated with the changes of EQ-5D-Y and PedsQL scores/subscales among those who completed both initial and latest follow-up assessments across times (T2-T1) (*n* = 83)

Variables	PedsQL															
	ΔPhysical Health				ΔEmotional				ΔSocial				ΔSchool			
	β*	95% CI		*p-value*	β*	95% CI		*p-value*	β*	95% CI		*p-value*	β*	95% CI		*p-value*
Intercept	98.15	93.60	102.70	< .0001	87.63	79.46	95.79	< .0001	96.43	92.95	99.91	< .0001	100.58	93.84	107.31	< .0001
Time (every 6 months)	0.68	0.40	0.96	< .0001	1.13	0.71	1.55	< .0001	0.51	0.26	0.77	< .0001	0.89	0.50	1.27	< .0001
Aged 13–18 years (vs 7–12)	-0.98	-3.17	1.21	0.3745	-0.19	-4.12	3.75	0.9249	2.14	0.47	3.81	0.0131	1.53	-1.72	4.78	0.3488
**Boys (vs Girls)**	0.08	-1.83	2.00	0.9299	-0.79	-4.26	2.69	0.6507	-0.52	-1.97	0.93	0.4732	**-3.17**	**-6.02**	**-0.31**	**0.0303**
Baseline eGFR	-0.02	-0.06	0.01	0.1889	0.01	-0.05	0.07	0.7127	-0.01	-0.03	0.02	0.5843	-0.07	-0.12	-0.02	0.0059
Change in eGFR, per month	**-1.76**	**-2.76**	**-0.76**	**0.0009**	-0.94	-2.72	0.84	0.2964	**-1.05**	**-1.83**	**-0.28**	**0.0084**	**-2.16**	**-3.64**	**-0.69**	**0.0048**
Number of comorbidities at T1(vs none)																
1	0.17	-3.58	3.91	0.9297	0.24	-6.52	7.01	0.9425	-0.79	-3.64	2.05	0.5785	-2.44	-8.00	3.13	0.3838
2	1.60	-4.67	7.88	0.6110	-3.96	-15.28	7.35	0.4857	-1.72	-6.50	3.05	0.4726	-8.23	-17.54	1.08	0.0821
≥ 3	-3.05	-13.20	7.09	0.5489	-10.67	-28.82	7.48	0.2435	-3.24	-11.04	4.56	0.4084	-12.70	-27.70	2.30	0.0954
Change in comorbidity (vs no–no at T1&T2)																
**Growth retardation**																
baseline ( +) & latest (-)	2.65	-3.00	8.30	0.3514	6.59	-3.67	16.85	0.2032	6.23	1.97	10.48	0.0049	0.55	-7.86	8.97	0.8959
baseline ( +) & latest ( +)	-3.88	-8.89	1.13	0.1267	3.62	-5.35	12.60	0.4217	-2.10	-5.95	1.75	0.2781	-3.73	-11.15	3.68	0.3172
**baseline (-) & latest ( +)**	-2.73	-6.31	0.84	0.1309	-0.71	-7.23	5.81	0.8281	-2.75	-5.43	**-0.07**	**0.0443**	-2.78	-8.11	2.55	0.3008
**Overweight**																
baseline ( +) & latest (-)	0.46	-4.76	5.69	0.8599	4.41	-4.91	13.73	0.3473	3.83	-0.20	7.87	0.0623	4.82	-2.89	12.54	0.2156
**baseline ( +) & latest ( +)**	3.22	-0.34	6.79	0.0756	**6.79**	**0.39**	**13.20**	**0.0381**	**3.10**	**0.38**	**5.83**	**0.0264**	**6.07**	**0.78**	**11.35**	**0.0252**
baseline (-) & latest ( +)	1.33	-2.07	4.72	0.4365	4.09	-2.07	10.26	0.1889	0.17	-2.38	2.72	0.8915	-1.17	-6.23	3.88	0.6432
**Hypertension**																
baseline ( +) & latest (-)	-1.49	-6.03	3.06	0.5151	4.37	-3.87	12.61	0.2927	1.91	-1.53	5.34	0.2713	4.75	-2.02	11.51	0.1653
baseline ( +) & latest ( +)	-1.00	-4.66	2.66	0.5872	-0.42	-6.99	6.14	0.8981	-0.74	-3.54	2.07	0.5997	-0.42	-5.84	5.00	0.8763
**baseline (-) & latest ( +)**	**-2.53**	**-4.91**	**-0.15**	**0.0375**	-2.45	-6.74	1.84	0.2575	0.24	-1.57	2.05	0.7923	**-4.26**	**-7.79**	**-0.73**	**0.0190**
**Hyperlipidemia**																
baseline ( +) & latest (-)	1.55	-5.66	8.76	0.6677	-3.27	-16.05	9.52	0.6104	2.94	-2.67	8.55	0.2975	-3.08	-13.70	7.53	0.5630
baseline ( +) & latest ( +)	-0.71	-4.51	3.10	0.7112	0.01	-6.87	6.88	0.9987	1.95	-0.93	4.84	0.1799	-0.91	-6.56	4.74	0.7484
baseline (-) & latest ( +)	-0.46	-4.51	3.59	0.8198	1.41	-5.91	8.74	0.7004	2.71	-0.36	5.78	0.0828	6.10	0.08	12.12	0.0470
**Mineral bone disorders/Anemia**																
baseline ( +) & latest (-)	-0.01	-5.00	4.98	0.9955	0.13	-8.90	9.15	0.9778	-0.30	-4.08	3.48	0.8745	-4.42	-11.84	3.00	0.2373
**baseline ( +) & latest ( +)**	**-6.88**	**-12.37**	**-1.40**	**0.0149**	-6.13	-16.00	3.74	0.2181	0.72	-3.46	4.90	0.7309	-12.90	-21.04	**-4.77**	**0.0024**
**baseline (-) & latest ( +)**	-2.68	-6.39	1.03	0.1530	-2.88	-9.58	3.81	0.3918	-2.43	-5.24	0.38	0.0884	-5.47	-10.98	**0.03**	**0.0513**
**Hyperuricemia**																
baseline ( +) & latest (-)	-3.79	-8.54	0.95	0.1150	1.49	-7.04	10.03	0.7270	0.82	-2.80	4.44	0.6525	0.53	-6.51	7.56	0.8807
baseline ( +) & latest ( +)	-1.37	-5.47	2.73	0.5049	4.35	-2.98	11.69	0.2395	-2.35	-5.50	0.80	0.1400	4.62	-1.45	10.68	0.1328
**baseline (-) & latest ( +)**	-2.25	-4.96	0.46	0.1022	-3.14	-8.05	1.76	0.2043	**-3.00**	**-5.05**	**-0.94**	**0.0050**	**-5.23**	**-9.26**	**-1.19**	**0.0120**
**Proteinuria**																
**baseline ( +) & latest (-)**	**-5.30**	**-9.64**	**-0.96**	**0.0177**	2.74	-5.07	10.54	0.4850	**-4.70**	**-8.01**	**-1.38**	**0.0063**	-1.56	-7.99	4.88	0.6300
baseline ( +) & latest ( +)	1.42	-2.53	5.38	0.4732	6.36	-0.76	13.47	0.0790	1.42	-1.60	4.43	0.3502	2.92	-2.94	8.78	0.3225
baseline (-) & latest ( +)	-2.31	-6.73	2.11	0.2995	3.95	-4.03	11.92	0.3252	-0.14	-3.49	3.21	0.9343	4.67	-1.89	11.23	0.1590

Δ: T2-T1 scores; β*: adjusted regression coefficient in a linear mixed model for each subscale of PedsQL change

Furthermore, the responsiveness of each PedsQL subscale score varied depending on the CKD-related comorbidity. For instance, declined physical health (adjusted β, -6.88, *p* = 0.0149) and school (adjusted β, -12.9, *p* = 0.0024) PedsQL scores were responsive to persistent mineral bone disorder/anemia; school functioning was responsive to developed hypertension and persistent mineral bone disorder/anemia (Tables 5 and 6). In addition, male patients had lower school PedsQL scores than female patients (adjusted β, -3.17, *p* = 0.0303).

## Discussion

This study aimed to investigate the concordance and responsiveness of HRQOL measures between EQ-5D-Y and PedsQL Generic score 4.0 in Taiwanese children and adolescents with mild-to-moderate CKD over time. Children and adolescents without comorbidity rated their VAS and LSS of EQ-5D-Y as equivalent to those with more comorbidity in their initial visits; similar results were applied for physical health and school domains of PedsQL. We observed both HRQOL instruments could detect the absolute difference in the number of CKD-related comorbidity over time. Furthermore, the associations between status changes in overweight, hypertension, hyperuricemia, and HRQOL changes were consistent between EQ-5D-Y and PedsQL measures.

Over 80% of children and adolescents reported VAS of EQ-5D-Y ≥ 90 in their initial visits (median VAS, 97 [IQR, 90, 100]). The high ceiling effects in this study cohort are consistent with those of previous studies that analyzed the same mild-to-moderate CKD cohort [10] and general population samples in Japan [28], Korea [30], and China [31, 32]. It seemed that children and adolescents with CKD felt relatively minor difficulties in “mobility” and “usual activities” on EQ-5D-Y (all participants responded “no problem”). Alternatively, EQ-5D-Y was based on 3-level ordinal response choices, which may not be ideal for measuring changes in children’s HRQO. Studies have found that EQ-5D-Y-5L can reduce the ceiling effect [33, 34] and had greater sensitivity discriminate ability than EQ-5D-Y-3L on pain/discomfort and doing usual activities domains in the pediatric orthopedic population [33].

Moreover, previous studies have indicated the low associations between EQ-5D-Y-3L dimension and PedsQL subscales scores in a cross-sectional fashion, such as in the pediatric orthopedic patients in South Africa [33], pediatric hematologic patients in Indonesia [35], and general population samples in Japan [28]. We found evident correlations (Spearman ρ > 0.3) between longitudinal changes in VAS of EQ-5D-Y and PedsQL for total score, emotional and social subscales (all Spearman ρ = 0.32, all *p* < 0.005), whereas no correlations (Spearman ρ < 0.3) appeared between each domain of EQ-5D-Y and PedsQL scores. The EQ-5D-Y did not include any environmental factors. Therefore, the VAS of EQ-5D-Y may partially reflect the daily activities of the school-aged children and adolescents’ social and school subscales, but not for each domain. Similarly, the LSS of EQ-5D-Y reflects the 5 domains of health state changes over time, but no correlation with PedsQL scores was observed (Table 3). When the Taiwan version of value set for the EQ-5D-Y is available, values for the health state changes over time may be observed in children with CKD.

This study has important clinical implications. When comparing those with no–no CKD-related comorbidity over time (initial-latest visit), the changed comorbidity status was associated with different degrees of longitudinal changes in VAS and LSS of EQ-5D-Y, and some domains of PedsQL scores. It was expected that the CKD-related comorbidity can persist for several years and has a negative impact on a child’s quality of life during the developmental ages. However, in the linear mixed regression analysis, some discordant associations between a comorbidity status and HRQOL changes appeared in this study cohort. For instance, persistent or developed overweight and hyperlipidemia were associated with increased VAS of EQ-5D-Y and some subscales of PedsQL scores during the follow-up visits. In addition, the increase in HRQOL score over time could be partly due to regular specific medical management at a specific time period, such as when corticosteroids are used to treat glomerular disorders but cause weight gain.

For the psychometric evaluation, Verstraete et al. have suggested that the VAS of EQ-5D-Y performed best in acutely-ill children to discriminate worst health status with the lowest VAS score of EQ-5D-Y than the total score of PedsQL in children and adolescents with acute health conditions [36]. It is also worth mentioning that EQ-5D-Y refers to the “today” health state using a three-level ordinal scale, whereas the five-point Likert scale in the PedsQL refers to the “past month”, which might have an impact on the divergence validity of these HRQOL measures [36, 37].

This is a cohort of pediatric patients with mild-to-moderate CKD. Children and adolescents with a longer duration of disease may have adapted their assessments of HRQOL according to their chronic disease status (e.g., persistent or no changes in some specific CKD comorbidity). These study findings indicate that further investigation of the discordance found in these domains of health associated with a particular treatment or management would enable a better understanding of how HRQOL changes in a particular health domain for CKD children. In addition, it is critical to ensure that HRQOL instruments have demonstrated sensitivity to detecting the changes in health perception and characteristics during a child’s development over time [38].

To our knowledge, we present the first comprehensive direct comparisons of EQ-5D-Y and PedsQL in contemporary pediatric clinical practice over time. This is the first test of the psychometric properties of the EQ-5D-Y and PedsQL in pediatric patients with mild-to-moderate CKD in cooperation with health status changes in longitudinal features. These study results highlight the necessity of long-term HRQOL assessment in children and adolescents with developmental changes in progressive CKD. Furthermore, this study demonstrated that both EQ-5D-Y and PedsQL could detect an evident difference in HRQOL scores according to the dynamic changes in CKD-related comorbid conditions.

It is worth mentioning that correlations between parent proxy-reported PedsQL scores and child self-reported EQ-5D-Y were very small, suggested that parents who rated HRQOL for their child’s health status were different from that of their children who were of the same aspects of HRQOL [18]. These results provide additional support for the importance of focusing on the child’s viewpoint rather than their parents' viewpoint for the ongoing assessment of HRQOL in children and adolescents with CKD. The proxy-version of EQ-5D-Y now is available and recommended for younger children (under the age of 7–8 years) and older children if they cannot complete the instrument themselves [39]. These study results may contribute to the growing evidence related to the measurement properties of both instruments in the pediatric CKD population. Further research is recommended comparing the psychometric performance of parent-proxy version of EQ-5D-Yand PedsQL to guide clinicians on the choice of most appropriate HRQOL instrument for the age-specific children and adolescents with CKD.

This study had some limitations. First, this single-center study included only limited patients who could attend our research clinic; therefore, the results may not be generalizable to the most affected children and adolescents with mild-to-moderate CKD. However, as age and prevalent clinical conditions in this study were similar to the patterns reported in multicenter studies [40], this limitation seems acceptable to the Taiwanese pediatric CKD population. Second, PMP-CKD is an observational study; thus, the specific treatment and management relevant to the mitigation of the severity of CKD-related comorbidity were not accounted for in the changes in HRQOL scores. Finally, we used four-level temporal clinical changes (i.e., persistent, developed, improved, and no change) in each comorbid condition to serve as a proxy of changes in disease burden in the specific period; future studies may be warranted to confirm these study findings.

In summary, this study demonstrated the small to moderate convergent validity between the EQ-5D-Y and PedsQL. The LSS of EQ-5D-Y generally showed a larger effect size than the VAS by the changes in the number of CKD-related comorbidity over time. Further comparisons of utility weights may help determine the responsiveness to changes between VAS and LSS of EQ-5D-Y. Therefore, both EQ-5D-Y and PedsQL can be considered as acceptable valid generic tools to assess HRQOL, reflecting longitudinal changes in HRQOL associated with complex CKD comorbidity management.

### Supplementary Information

**Additional file 1:** **Table S1.** Distribution of EQ-5D-Y domains at initial assessment. **Table S2.** VAS and LSS of EQ-5D-Y at baseline. **Table S3.** Cross-sectional correlations between EQ-5D-Y and PedsQL scores at initial and latest follow-up assessments. **Table S4.** Correlations between changes in children’s self-reported EQ-5D-Y and changes in parent-proxy reported PedsQL scores. **Table S5.** Characteristics of study participants in longitudinal analysis (*n* = 83).

## Data Availability

The data are not available. According to the informed consent content, participants provided raw data would remain confidential and would not be shared.
