# Supplementary material for: Comparisons of EQ-5D-Y and PedsQL in pediatric patients with mild-to-moderate chronic kidney disease in longitudinal analyses
Source: Health Qual Life Outcomes. 2023 Oct 27;21:117. doi: 10.1186/s12955-023-02197-9 (PMC10605985; doi:10.1186/s12955-023-02197-9)
Supplement: Supplementary file 1 — Additional file 1: Table S1. Distribution of EQ-5D-Y domains at initial assessment. Table S2. VAS and LSS of EQ-5D-Y at baseline. Table S3. Cross-sectional correlations between EQ-5D-Y and PedsQL scores at initial and latest follow-up assessments. Table S4. Correlations between changes in children’s self-reported EQ-5D-Y and changes in parent-proxy reported PedsQL scores. Table S5. Characteristics of study participants in longitudinal analysis (n = 83). [file 12955_2023_2197_MOESM1_ESM.docx]

Comparisons of EQ-5D-Y and PedsQL in pediatric patients with mild-to-moderate chronic kidney disease in longitudinal analyses

**Supplementary Material**

Table S1. Distribution of EQ-5D-Y scores and responses on each domain among all enrolled pediatric patients in their initial visits

Table S2. The distribution of EQ-5D-Y VAS grouped as the levels of LSS among all enrolled pediatric patients in their initial visits

Table S3. Cross-sectional correlations between EQ-5D-Y and PedsQL scores/subscales in the initial and latest follow-up visits

Table S4. Correlations between changes in children self-reported EQ-5D-Y and changes in parent-proxy reported PedsQL scores across times among those who completed both initial and latest follow-up assessments (n=83)

Table S5. Characteristics of study cohort who completed both initial and latest follow-up assessments categorized based on their changes on numbers of CKD-related comorbidities (n=83)

Table S1 Distribution of EQ-5D-Y scores and responses on each domain among all enrolled pediatric patients in their initial visits

| **EQ-5D-Y** | | | | | All pediatric participants (n=121) | | | | |  | Younger children  7-12 years  (n=68) | | | | |  | Adolescent children  13-18 years  (n=53) | | | | *P value^a^* | |  |
| --- | --- | --- | --- | --- | --- | --- | --- | --- | --- | --- | --- | --- | --- | --- | --- | --- | --- | --- | --- | --- | --- | --- | --- |
| **Mobility, n(%)** | | | | |  | |  | | |  |  | |  | | |  |  | |  | | 0.7113 | |  |
|  | | No problem | | | 118 | | (97.3) | | |  | 66 | | (97.1) | | |  | 52 | | (98.1) | |  | |  |
|  | | Have any problems | | | 3 | | (2.5) | | |  | 2 | | (2.9) | | |  | 1 | | (1.90) | |  | |  |
|  | |  | Some problems | | 3 | | (2.5) | | |  | 2 | | (2.9) | | |  | 1 | | (1.90) | |  | |  |
|  | |  | A lot of problems | | 0 | |  | | |  | 0 | |  | | |  | 0 | |  | |  | |  |
| **Self-care, n(%)** | | | | |  | |  | | |  |  | |  | | |  |  | |  | | - | |  |
|  | | No problem | | | 121 | | (100.0) | | |  | 68 | | (100.0) | | |  | 53 | | (100.0) | |  | |  |
|  | | Have any problems | | | 0 | |  | | |  | 0 | |  | | |  | 0 | |  | |  | |  |
| **Usual activities, n(%)** | | | | |  | |  | | |  |  | |  | | |  |  | |  | | - | |  |
|  | | No problem | | | 121 | | (100.0) | | |  | 68 | | (100.0) | | |  | 53 | | (100.0) | |  | |  |
|  | | Have any problems | | | 0 | |  | | |  | 0 | |  | | |  | 0 | |  | |  | |  |
| **Pain/discomfort, n(%)** | | | | |  | |  | | |  |  | |  | | |  |  | |  | | 0.0471 | |  |
|  | | No problem | | | 106 | | (87.6) | | |  | 56 | | (82.4) | | |  | 50 | | (94.3) | |  | |  |
|  | | Have any problems | | | 15 | | (12.4) | | |  | 12 | | (17.7) | | |  | 3 | | (5.7) | |  | |  |
|  | |  | Some problems | | 15 | | (12.4) | | |  | 12 | | (17.7) | | |  | 3 | | (5.7) | |  | |  |
|  | |  | A lot of problems | | 0 | |  | | |  | 0 | |  | | |  | 0 | |  | |  | |  |
| **Anxiety/depression, n(%)** | | | | |  | |  | | |  |  | |  | | |  |  | |  | | 0.3161 | |  |
|  | | No problem | | | 108 | | (89.3) | | |  | 59 | | (86.8) | | |  | 49 | | (92.5) | |  | |  |
|  | | Have any problems | | | 13 | | (10.7) | | |  | 9 | | (13.2) | | |  | 4 | | (7.6) | |  | |  |
|  | |  | Some problems | | 13 | | (10.7) | | |  | 9 | | (13.2) | | |  | 4 | | (7.6) | |  | |  |
|  | |  | A lot of problems | | 0 | |  | | |  | 0 | |  | | |  | 0 | |  | |  | |  |
| **VAS** | | | | |  | |  | | |  |  | | |  | |  |  | |  | |  | |  |
|  | Mean (SD) | | | 93.3 | | (11.6) | |  | 95.5 | | | (7.6) | | | 90.6 | | | (15.0) | | 0.0322 | |  |  |
|  | Median (25^th^,75^th^) | | | 97.0 | | (90.0, 100.0) | |  | 100.0 | | | (95.0, 100.0) | | | 95.0 | | | (90.0, 100.0) | | 0.0026 | |  |  |
| **LSS** | | | | | | | | | | | | | | | | | | | | | | | |
|  | Mean (SD) | | | 5.3 | | (0.6) | |  | 5.3 | | | (0.6) | | | 5.2 | | | (0.5) | | 0.0656 | |  |  |
|  | Median ((25^th^,75^th^) | | | 5.0 | | (5.00, 6.0) | |  | 5.0 | | | (5.0, 6.0) | | | 5.0 | | | (5.0, 5.0) | | 0.0261 | |  |  |

^a^ Comparison of different age group by Chi-square test or Fisher's exact test for categorical data; Independent t test or Wilcoxon rank sum test for continuous data .

VAS, Visual Analogue Scale; LSS, Level Sum Score

Table S2 The distribution of EQ-5D-Y VAS grouped as the levels of LSS among all enrolled pediatric patients in their initial visits

|  |  | VAS of EQ-5D-Y | | | | |
| --- | --- | --- | --- | --- | --- | --- |
|  |  | Number of patients | Mean | (SD) | Median | (25^th^,75^th^) |
| **LSS of EQ-5D-Y** | | |  |  |  |  |
|  | 5 | 96 | 93.63 | (8.72) | 95.00 | (90.00, 100.00) |
|  | 6-8 | 25 | 92.12 | (19.28) | 97.00 | (95.00, 100.00) |
| **LSS =7** | |  |  |  |  |  |
|  | 11122 | 4 | 98.00 | (2.45) | 98.50 | (96.00, 100.00) |
| **LSS =6** | |  |  |  |  |  |
|  | 11112 | 8 | 92.13 | (10.09) | 93.50 | (90.00, 100.00) |
|  | 11121 | 10 | 97.40 | (3.47) | 99.50 | (95.00, 100.00) |
|  | 21111 | 2 | 97.50 | (3.54) | 97.50 | (95.00, 100.00) |

VAS, Visual Analogue Scale; LSS, Level Sum Score; SD, standard deviation

Table S3 Cross-sectional correlations between EQ-5D-Y and PedsQL scores/subscales in the initial and latest follow-up visits

| **PedsQL** | **EQ-5D-Y** | | | | | | | | | | | | | |
| --- | --- | --- | --- | --- | --- | --- | --- | --- | --- | --- | --- | --- | --- | --- |
|  | **EQ-5D-Y-VAS** | |  | **EQ-5D-Y-LSS** | |  | **Mobility** | |  | **Pain/discomfort** | |  | **Anxiety/depression** | |
|  | ρ | p-value |  | ρ | p-value |  | ρ | p-value |  | ρ | p-value |  | ρ | p-value |
| **Initial visit (n=121)** |  | | | | | | | | | | | | | |
| Total Score | 0.25 | 0.0067 |  | -0.18 | 0.0459 |  | -0.05 | 0.5498 |  | -0.17 | 0.0660 |  | -0.14 | 0.1232 |
| Physical Health | 0.12 | 0.2060 |  | -0.17 | 0.0591 |  | -0.05 | 0.6198 |  | -0.20 | 0.0286 |  | -0.11 | 0.2215 |
| Emotional | 0.24 | 0.0081 |  | -0.17 | 0.0597 |  | -0.02 | 0.8551 |  | -0.11 | 0.2476 |  | -0.17 | 0.0591 |
| Social | 0.06 | 0.5279 |  | -0.12 | 0.2050 |  | 0.01 | 0.8939 |  | -0.10 | 0.2729 |  | -0.07 | 0.4454 |
| School | 0.24 | 0.0088 |  | -0.12 | 0.1767 |  | -0.07 | 0.4698 |  | -0.12 | 0.1909 |  | -0.06 | 0.5268 |
| **Latest follow-up visit (n=83)** | | | | | | | | | | | | | | |
| Total Score | 0.38 | 0.0004 |  | -0.31 | 0.0039 |  | -0.17 | 0.1198 |  | -0.21 | 0.0546 |  | -0.18 | 0.1033 |
| Physical Health | 0.40 | 0.0002 |  | -0.27 | 0.0120 |  | -0.21 | 0.0510 |  | -0.18 | 0.0987 |  | -0.21 | 0.0575 |
| Emotional | 0.28 | 0.0097 |  | -0.39 | 0.0003 |  | -0.20 | 0.0677 |  | -0.21 | 0.0568 |  | -0.28 | 0.0094 |
| Social | 0.28 | 0.0104 |  | -0.23 | 0.0362 |  | -0.27 | 0.0123 |  | -0.11 | 0.3338 |  | -0.05 | 0.6645 |
| School | 0.22 | 0.0487 |  | -0.16 | 0.1361 |  | -0.07 | 0.5349 |  | -0.16 | 0.1611 |  | -0.03 | 0.8107 |

ρ (Rho): Spearman's correlation coefficient, positive correlation coefficient indicates agreement between measures; negative correlation coefficient indicates disagreement between measures.

VAS, Visual Analogue Scale; LSS, Level Sum Score, A higher LSS indicated a worse health state.

Table S4 Correlations between changes in children self-reported EQ-5D-Y and changes in parent-proxy reported PedsQL scores across times among those who completed both initial and latest follow-up assessments (n=83)

|  | Absolute difference in summary scores of EQ-5D-Y | | | | |  | Change in each dimension  of EQ-5D-Y | | | | | | | | | | | | | | | | |  |  |
| --- | --- | --- | --- | --- | --- | --- | --- | --- | --- | --- | --- | --- | --- | --- | --- | --- | --- | --- | --- | --- | --- | --- | --- | --- | --- |
| Difference in PedsQL | VAS | |  | LSS | |  | Mobility | |  | Usual activities | | |  | Pain/discomfort | | | | | |  | Anxiety/depression | | | | |
|  | ρ | p-value |  | ρ | p-value |  | ρ | p-value |  | ρ | p-value | |  | ρ | | p-value | | | |  | ρ | | p-value | | |
| Total Score | 0.09 | 0.3772 |  | -0.06 | 0.5783 |  | 0.11 | 0.2945 |  | -0.16 | | 0.1387 |  | | -0.01 | | 0.9312 |  | -0.08 | | | 0.4621 | | |  |
| Physical Health | 0.01 | 0.9280 |  | -0.06 | 0.5673 |  | 0.04 | 0.7162 |  | -0.14 | | 0.1901 |  | | -0.11 | | 0.3095 |  | -0.05 | | | 0.6221 | | |  |
| Emotional | 0.17 | 0.1109 |  | -0.10 | 0.3729 |  | 0.07 | 0.5059 |  | 0.02 | | 0.8288 |  | | 0.02 | | 0.8386 |  | -0.19 | | | 0.0706 | | |  |
| Social | 0.13 | 0.2255 |  | 0.05 | 0.6410 |  | 0.23 | 0.0314 |  | -0.19 | | 0.0804 |  | | 0.06 | | 0.5756 |  | 0.04 | | | 0.7297 | | |  |
| School | 0.13 | 0.2104 |  | -0.03 | 0.8088 |  | 0.06 | 0.5898 |  | -0.08 | | 0.4268 |  | | 0.02 | | 0.8451 |  | -0.06 | | | 0.5887 | | |  |

“Self-care” of EQ-5D-Y is not presented due to the no patients reported any problems at initial (T1) and the latest follow-up assessments (T2);

Difference= T2 score minus T1 score; 3-level change in each dimension of EQ-5D-Y, including improve (T1: having any problem, T2: no problem), no change (T1 and T2 same), and worst (T1: no problem, T2: having any problem)

ρ (Rho): Spearman's correlation coefficient, positive correlation coefficient indicates agreement between measures; negative correlation coefficient indicates disagreement between measures.

VAS, Visual Analogue Scale; LSS, Level Sum Score, A higher LSS indicated a worse health state.

Table S5 Characteristics of study cohort who completed both initial and latest follow-up assessments categorized based on their changes on numbers of CKD-related comorbidities (n=83)

|  | | | **Number of comorbidities** | | | | | | | | | | | | | | | | |  | |  |  |  |
| --- | --- | --- | --- | --- | --- | --- | --- | --- | --- | --- | --- | --- | --- | --- | --- | --- | --- | --- | --- | --- | --- | --- | --- | --- |
| **Characteristics** | | | **No change**  (N=34) | | |  | | **Improve**  (N=16) | | | |  | | Worsen  (N=33) | | | | | | *P-value^a^* | |  |  |  |
|  |  |  | n | (%) | |  | | n | (%) | | |  | | n | | (%) | | | |  |  |  |  |  |
| **Age group, year, n(%)** | | |  |  | |  | |  |  | | |  | |  | |  | | | | 0.5671 | |  |  |  |
|  | 7-12 | | 18 | (52.94) | |  | | 8 | (50.00) | | |  | | 21 | | (63.64) | | | | . | |  |  |  |
|  | 13-18 | | 16 | (47.06) | |  | | 8 | (50.00) | | |  | | 12 | | (36.36) | | | |  | |  |  |  |
| **Sex, n(%)** | | |  |  | |  | |  |  | | |  | |  | |  | | | | 0.8321 | |  |  |  |
|  | Boy | | 20 | (58.82) | |  | | 9 | (56.25) | | |  | | 17 | | (51.52) | | | | . | |  |  |  |
|  | Girl | | 14 | (41.18) | |  | | 7 | (43.75) | | |  | | 16 | | (48.48) | | | |  | |  |  |  |
| **Type of CKD, n(%)** | | |  | |  | |  | | |  |  | | | |  | | | |  |  | 0.2177 |  | | |
|  | 1.CAKUT_Q60 | | 10 | (29.41) | |  | | 8 | (50.00) | | |  | | 17 | | (51.52) | | | |  | |  |  |  |
|  | 2.CAKUT_ other | | 6 | (17.65) | |  | | 4 | (25.00) | | |  | | 3 | | (9.09) | | | | . | |  |  |  |
|  | 3.GD | | 11 | (32.35) | |  | | 3 | (18.75) | | |  | | 5 | | (15.15) | | | | . | |  |  |  |
|  | 4.CKD/SLE/Others | | 7 | (20.59) | |  | | 1 | (6.25) | | |  | | 8 | | (24.24) | | | | . | |  |  |  |
| **Baseline eGFR,** ml/min/1.73m2 | | | 107.53 | (34.15) | |  | | 96.67 | (16.60) | | |  | | 108.73 | | (42.82) | | | | 0.5104 | |  |  |  |
| **Level of eGFR at baseline, n(%)** | | | |  | |  | |  |  | | |  | |  | |  | | | | 0.6486 | |  |  |  |
|  | >= 90 | | 22 | (64.71) | |  | | 11 | (68.75) | | |  | | 24 | | (72.73) | | | |  | |  |  |  |
|  | 60-89 | | 9 | (26.47) | |  | | 5 | (31.25) | | |  | | 8 | | (24.24) | | | | . | |  |  |  |
|  | 30-59 | | 3 | (8.82) | |  | | 0 | (0.00) | | |  | | 1 | | (3.03) | | | | . | |  |  |  |
| Growth retardation, **n(%)** | | | 7 | (20.59) | |  | | 3 | (18.75) | | |  | | 2 | | (6.06) | | | | 0.2067 | |  |  |  |
| Overweight, **n(%)** | | | 12 | (35.29) | |  | | 5 | (31.25) | | |  | | 8 | | (24.24) | | | | 0.6115 | |  |  |  |
| Hypertension, **n(%)** | | | 17 | (50.00) | |  | | 6 | (37.50) | | |  | | 5 | | (15.15) | | | | 0.0099 | |  |  |  |
| Hyperlipidemia, **n(%)** | | | 7 | (20.59) | |  | | 2 | (12.50) | | |  | | 5 | | (15.15) | | | | 0.7325 | |  |  |  |
| Mineral bone disorders/Anemia, **n(%)** | | | 5 | (14.71) | |  | | 5 | (31.25) | | |  | | 1 | | (3.03) | | | | 0.0227 | |  |  |  |
| Hyperuricemia, **n(%)** | | | 9 | (26.47) | |  | | 5 | (31.25) | | |  | | 3 | | (9.09) | | | | 0.1045 | |  |  |  |
| Proteinuria, **n(%)** | | | 12 | (35.29) | |  | | 6 | (37.50) | | |  | | 7 | | (21.21) | | | | 0.3516 | |  |  |  |
| **Number of comorbid conditions in initial visits, n(%)** | | | | | | | | | | | | | | | | | | | | **0.0004** | |  |  |  |
|  | None | | 7 | (20.59) | |  | | 0 | (0.00) | | |  | | 12 | | (36.36) | | | |  | |  |  |  |
|  | 1 |  | 7 | (20.59) | |  | | 4 | (25.00) | | |  | | 11 | | (33.33) | | | | . | |  |  |  |
|  | 2 |  | 6 | (17.65) | |  | | 8 | (50.00) | | |  | | 10 | | (30.30) | | | | . | |  |  |  |
|  | >=3 | | 14 | (41.18) | |  | | 4 | (25.00) | | |  | | 0 | | (0.00) | | | | . | |  |  |  |
| **Change in comorbid condition between T1 and T2** | | | | | | | | | | | | |  | | | |  |  | | |  | |  |  |
| **Mean change in eGFR, per month** | | | -0.45 | (0.82) | |  | | 0.28 | (1.10) | | |  | | -0.56 | | (1.40) | | | | 0.0493 | |  |  |  |
| **Level of eGFR, n(%)** | | |  |  | |  | |  |  | | |  | |  | |  | | | | 0.7091 | |  |  |  |
|  | No change | | 28 | (82.35) | |  | | 11 | (68.75) | | |  | | 23 | | (69.70) | | | |  | |  |  |  |
|  | Improved | | 2 | (5.88) | |  | | 1 | (6.25) | | |  | | 2 | | (6.06) | | | | . | |  |  |  |
|  | Worsened | | 4 | (11.76) | |  | | 4 | (25.00) | | |  | | 8 | | (24.24) | | | | . | |  |  |  |
| **Growth retardation, n(%)** | | |  |  | |  | |  |  | | |  | |  | |  | | | | 0.2744 | |  |  |  |
|  | No change | | 30 | (88.24) | |  | | 14 | (87.50) | | |  | | 30 | | (90.91) | | | | . | |  |  |  |
|  | Improved | | 2 | (5.88) | |  | | 2 | (12.50) | | |  | | 0 | | (0.00) | | | | . | |  |  |  |
|  | Worsened | | 2 | (5.88) | |  | | 0 | (0.00) | | |  | | 3 | | (9.09) | | | |  | |  |  |  |
| **Overweight, n(%)** | | |  |  | |  | |  |  | | |  | |  | |  | | | | 0.0053 | |  |  |  |
|  | No change | | 31 | (91.18) | |  | | 13 | (81.25) | | |  | | 27 | | (81.82) | | | | . | |  |  |  |
|  | Improved | | 3 | (8.82) | |  | | 3 | (18.75) | | |  | | 0 | | (0.00) | | | | . | |  |  |  |
|  | Worsened | | 0 | (0.00) | |  | | 0 | (0.00) | | |  | | 6 | | (18.18) | | | |  | |  |  |  |
| **Hypertension, n(%)** | | |  |  | |  | |  |  | | |  | |  | |  | | | | 0.0001 | |  |  |  |
|  | No change | | 26 | (76.47) | |  | | 12 | (75.00) | | |  | | 12 | | (36.36) | | | | . | |  |  |  |
|  | Improved | | 2 | (5.88) | |  | | 3 | (18.75) | | |  | | 1 | | (3.03) | | | | . | |  |  |  |
|  | Worsened | | 6 | (17.65) | |  | | 1 | (6.25) | | |  | | 20 | | (60.61) | | | |  | |  |  |  |
| **Hyperlipidemia, n(%)** | | |  |  | |  | |  |  | | |  | |  | |  | | | | 0.3804 | |  |  |  |
|  | No change | | 31 | (91.18) | |  | | 15 | (93.75) | | |  | | 29 | | (87.88) | | | | . | |  |  |  |
|  | Improved | | 1 | (2.94) | |  | | 1 | (6.25) | | |  | | 0 | | (0.00) | | | | . | |  |  |  |
|  | Worsened | | 2 | (5.88) | |  | | 0 | (0.00) | | |  | | 4 | | (12.12) | | | |  | |  |  |  |
| **Mineral bone disorders/Anemia, n(%)** | | | |  | |  | |  |  | | |  | |  | |  | | | | 0.0142 | |  |  |  |
|  | No change | | 30 | (88.24) | |  | | 12 | (75.00) | | |  | | 26 | | (78.79) | | | | . | |  |  |  |
|  | Improved | | 1 | (2.94) | |  | | 4 | (25.00) | | |  | | 1 | | (3.03) | | | | . | |  |  |  |
|  | Worsened | | 3 | (8.82) | |  | | 0 | (0.00) | | |  | | 6 | | (18.18) | | | |  | |  |  |  |
| **Hyperuricemia, n(%)** | | |  |  | |  | |  |  | | |  | |  | |  | | | | 0.0028 | |  |  |  |
|  | No change | | 29 | (85.29) | |  | | 12 | (75.00) | | |  | | 22 | | (66.67) | | | | . | |  |  |  |
|  | Improved | | 1 | (2.94) | |  | | 4 | (25.00) | | |  | | 1 | | (3.03) | | | | . | |  |  |  |
|  | Worsened | | 4 | (11.76) | |  | | 0 | (0.00) | | |  | | 10 | | (30.30) | | | |  | |  |  |  |
| **Proteinuria n(%)** | | |  |  | |  | |  |  | | |  | |  | |  | | | | 0.1927 | |  |  |  |
|  | No change | | 28 | (82.35) | |  | | 12 | (75.00) | | |  | | 29 | | (87.88) | | | |  | |  |  |  |
|  | Improved | | 3 | (8.82) | |  | | 3 | (18.75) | | |  | | 0 | | (0.00) | | | | . | |  |  |  |
|  | Worsened | | 3 | (8.82) | |  | | 1 | (6.25) | | |  | | 4 | | (12.12) | | | | . | |  |  |  |

* Comparison of different clinical change groups by McNemer test on paired nominal data

No change: number of comorbidities was same at study enrollment (T1) and latest follow-up visit (T2); improved= number of comorbidities at T1 > T2; worsened= number of comorbidities at T1 < T2.
